# The RecQ helicase Sgs1 drives ATP-dependent disruption of Rad51 filaments

**DOI:** 10.1093/nar/gkz186

**Published:** 2019-03-27

**Authors:** J Brooks Crickard, Chaoyou Xue, Weibin Wang, Youngho Kwon, Patrick Sung, Eric C Greene

**Affiliations:** 1 Department of Biochemistry & Molecular Biophysics, Columbia University, New York, NY 10032, USA; 2 Department of Molecular Biophysics and Biochemistry, Yale University School of Medicine, New Haven, CT 06520, USA; 3 Department of Biochemistry and Structural Biology, University of Texas Health Science Center at San Antonio, TX 78229, USA

## Abstract

DNA helicases of the RecQ family are conserved among the three domains of life and play essential roles in genome maintenance. Mutations in several human RecQ helicases lead to diseases that are marked by cancer predisposition. The *Saccharomyces cerevisiae* RecQ helicase Sgs1 is orthologous to human BLM, defects in which cause the cancer-prone Bloom's Syndrome. Here, we use single–molecule imaging to provide a quantitative mechanistic understanding of Sgs1 activities on single stranded DNA (ssDNA), which is a central intermediate in all aspects of DNA metabolism. We show that Sgs1 acts upon ssDNA bound by either replication protein A (RPA) or the recombinase Rad51. Surprisingly, we find that Sgs1 utilizes a novel motor mechanism for disrupting ssDNA intermediates bound by the recombinase protein Rad51. The ability of Sgs1 to disrupt Rad51–ssDNA filaments may explain some of the defects engendered by RECQ helicase deficiencies in human cells.

## INTRODUCTION

RecQ helicases constitute a unique subgroup of the SF2 (super-family 2) of helicases and they play essential roles in the maintenance of genome integrity (1–6). Humans possess five RecQ homologs, namely WRN, BLM, RECQ1, RECQ4 and RECQ5 (1–6). Mutations in BLM, WRN, and RECQ4 cause Bloom, Werner, and Rothmund–Thompson syndromes, respectively, which are associated with profound developmental abnormalities and increased cancer risk, and the latter two syndromes are also characterized by premature ageing (1–4,6). The average Bloom syndrome patient lifespan is only 27 years and cancer is the leading cause of death (7–9). Cells from patients with Bloom Syndrome (BS) are marked by DNA damage hypersensitivity, elevated genome instability, and a ∼10-fold increase in sister chromatid exchanges (SCEs) (6–9). The SCE phenotype of BLM deficient cells reflects a failure to suppress crossover formation during homologous recombination (6–9).

Efforts to more fully understand the roles of BLM and other human RECQ helicases in the maintenance of genome integrity are confounded by the partial functional overlap of these proteins (1–5). Importantly, *Saccharomyces cerevisiae* Sgs1 is orthologous to human BLM and *sgs1Δ* mutations phenocopy many of the genome integrity defects observed in cells from Bloom syndrome patients (1,10). Indeed, yeast *sgs1Δ* mutants are extremely sensitive to DNA damaging agents, exhibit a reduced life span, frequent chromosome mis-segregation and extensive chromosomal rearrangements that likely stem from elevated crossover recombination events (1,10–13). Importantly, expression of human BLM or WRN in yeast partially rescues many of the *sgs1Δ* phenotypes (14).

RecQ helicases have been implicated the rescue of stalled or collapsed replication forks (1–6). Notably, Sgs1 and WRN both associate with unperturbed replication forks (15,16), and BLM is recruited to stalled replication forks (17). BLM and Sgs1 are also indispensable for chromosome damage repair by homologous recombination (HR), and have been implicated in numerous HR-related processes, including DNA end processing, suppression of illegitimate recombination, synthesis-dependent strand annealing (SDSA) and Holliday junction dissolution (1–6,11,18). Given the involvement of RecQ helicases in diverse nuclear processes, it is not surprising that *RECQ* mutations engender complex phenotypes, which complicates the understanding of their molecular functions.

To help delineate the roles of RecQ helicases in genome maintenance, here we use single-molecule imaging to visualize the behaviors of Sgs1 on ssDNA. We show that Sgs1 is capable of translocating over long distances along ssDNA that is bound by either replication protein A (RPA) or the recombinase Rad51. Surprisingly, our findings demonstrate that Sgs1 can translocate on ssDNA without removing RPA. In contrast, Sgs1 readily dismantles Rad51 filaments using a mechanism that is not coupled to the Rad51 ATP hydrolysis cycle. These findings indicate that Sgs1 acts through a mechanism that is fundamentally distinct from the second major antirecombinase in yeast, the SF1 helicase Srs2 (12,19–21). Importantly, we show that Sgs1 cannot act upon presynaptic filaments composed of the meiosis-specific recombinase Dmc1. Together, our findings provide new insights into the function of the Sgs1 ssDNA motor activity in mitotic DNA repair and have implications for understanding the mechanisms that help ensure optimal regulation of crossover recombination events in meiotic cells.

## MATERIALS AND METHODS

### Protein purification

*Saccharomyces cerevisiae* RPA, GFP-RPA, mCherry-RPA, Rad51, were purified as previously described (22–24). Sgs1 and GFP–Sgs1 were also purified as previously described (25). Briefly, Flag-His_6_-Sgs1 or Flag-His_6_-GFP–Sgs1 was expressed in insect cells. All purification steps were carried out at 4°C. The insect pellet was resuspended in K buffer (20 mM KH_2_PO_4_, 10% glycerol, 0.5 mM EDTA, 0.01% Igepal, 1 mM DTT) with (aprotonin, chymostatin, leupeptin and pepstatin at 5 μg/ml, and 1 mM phenyl-methylsulfonyl fluoride) and 500 mM KCl. Cells were lysed by sonication and clarified by ultracentrifuge at 100 000 × g for 45 min. The clarified extract was incubated with 1 ml of anti-FLAG M2 resin for 2 h. The resin was washed with K buffer with 500 mM KCl and 2 mM ATP, 2 mM MgCl_2_. The protein was eluted in 500 mM KCl and 2 mM ATP, 2 mM MgCl_2_ plus 200 μ/ml FLAG peptide. The eluate was the incubated in Buffer K plus 500 mM KCl, 2 mM ATP, 2 mM MgCl_2_ and 15 mM Imidazole with 300 μl of nickel-NTA resin. The protein was eluted in the same buffer plus 200 mM Imidazole. The imidazole was removed by filter dialysis, and the protein was concentrated down to 200 μg/ml, and stored at –80°C.

### ATP hydrolysis assays

ATP hydrolysis assays were performed in reaction buffer (30 mM Tris–Cl [pH 7.5], 100 mM KCl, 5 mM MgCl_2_, 1.5 mM CaCl_2_, 1 mM DTT, 0.2 mg/ml BSA) in the presence of M13 ssDNA (2.5 μM nucleotides total concentration) (NEB, Cat. No. N4040) 2 mM ATP and trace amounts of γ^32^P-ATP (3000 Ci/mmol). All reactions were performed at 30°C. Aliquots were removed at specified time points and quenched by mixing with an equal volume of 25 mM EDTA and 1% SDS. The quenched reactions were spotted on TLC plates (Millipore, Cat. No. HX71732079) and resolved in 0.5 M LiCl plus 1 M Formic acid. Dried TLC plates were exposed to phosphor-imaging screen, and scanned with a Typhoon platform (GE Healthcare). Note that the ATP hydrolysis activity contributed by Rad51 is insignificant compared to that of Sgs1: the reported *k*_cat_ for ATP hydrolysis for yeast Rad51 bound to ssDNA is 0.012 s^–1^ (26), whereas the reported *k*_cat_ for yeast Sgs1 in the presence of ssDNA is 256 ± 6 s^–1^ (27). Based on these literature values, the ATP hydrolysis activity is approximately 2 × 10^4^ times higher than that of Rad51.

### Single molecule data collection

All experiments were conducted with a prism-type total internal reflection fluorescence (TIRF) microscope (Nikon) equipped with a 488-nm laser (Coherent Sapphire, 200 mW), a 561-nm laser (Coherent Sapphire, 200 mW), and two Andor iXon EMCCD cameras (28,29). Flowcells and ssDNA curtains were prepared as previously described (28,29). In brief, lipid bilayers were prepared with 91.5% DOPC, 0.5% biotinylated-PE and 8% mPEG 2000-DOPE. The ssDNA substrate was generated using rolling circle replication with a biotinylated primer, a circular M13 ssDNA template, and Phi29 DNA polymerase, as described (28,29). The biotinylated ssDNA was injected into the sample chamber and attached to the bilayer through a biotin–streptavidin linkage. The flow cell was then attached to a microfluidic system and sample delivery was controlled using a syringe pump (Kd Scientific) (28,29). For all two-color images, we used a custom-built shuttering system to avoid signal bleed-through during image acquisition. With this system, images from the green (GFP) and the red (mCherry) channels are recorded independently, these recordings are offset by 100 ms such that when one camera records the red channel image, the green laser is shuttered off and vice versa (28,29).

### Recombinase filament assembly

ssDNA molecules were aligned along the diffusion barriers at a flow rate of 0.5 ml/min in reactions buffer plus RPA (30 mM Tris–Cl [pH 7.5], 100 mM KCl, 5 mM MgCl_2_, 1.5 mM CaCl_2_, 0.2 mg/ml BSA, 1 mM DTT, 0.1 nM RPA-GFP, RPA–mCherry or unlabeled RPA, as indicated). Once the ssDNA molecules were aligned, the flow rate was adjusted to 1.0 ml/min and 0.5 ml of 7 M urea was injected into the flow cell to help disrupt any remaining secondary structure. The sample chamber was then flushed with reaction buffer plus RPA-GFP or RPA–mCherry (0.1 nM) at 1.0 ml/min for 10 min. After 5 min, reaction buffer plus 2.5 mM ATP was flushed through the sample chamber at a flow rate of 1.0 ml/min for 3 min. Either Rad51 (2 μM) or Dmc1 (2 μM) was injected into the flow cell, buffer flow was terminated, and the reactions were incubated at 30°C for 20 min, and the RPA fluorescence signal was monitored to verify filament assembly. Free recombinase was then flushed from the sample chamber with reaction buffer plus 2.5 mM ATP.

### Sgs1 translocation assays and data analysis

All Sgs1 measurements were conducted at 30°C in reaction buffer supplemented with RPA (unlabeled, GFP-tagged or mCherry-tagged, as indicated) and 2.5 mM ATP. Samples containing either 10 nM GFP–Sgs1 plus 0.1 nM RPA–mCherry or 10 nM unlabeled Sgs1 plus 0.1 nM RPA-GFP were injected into the flow cell at a rate of 1.0 ml/min, flow then was stopped and the activity of Sgs1 was monitored for 20–25 min. All data were collected as previously described for Srs2 (29,30). In brief, images with captured at an acquisition rate of 1 frame per 10 s with a 200-ms integration time, and the laser was shuttered between each acquired image to minimize photo-bleaching. Raw TIFF images were imported as image stacks into ImageJ, and kymographs were generated from the image stacks by defining a 1-pixel wide region of interest (ROI) along the long-axis of the individual ssDNA molecules. Sgs1 translocation velocity was calculated from the kymographs by manually measuring the distance travelled as a function of time. The velocities were then plotted 10 nt/s bins and the resulting histograms were fit to a Gaussian distribution using Prism 7 (Graphpad Software, Inc.). Reported velocities represent the mean ± the standard deviation generated from these fits. For Sgs1 processivity, the distance a molecule traveled was calculated in pixels, the distances values where changed nucleotides using a conversion factor of 1000 nt/pixel, and the resulting data were used to generate survival plots, as described (29,30). The survival plots were fit as single exponential decay curves, and the reported processivity values corresponds to the half-life obtained from these curves. Error bars were generated by bootstrapping using a custom python script.

## RESULTS

### Sgs1 can translocate rapidly on RPA-bound ssDNA

**Figure 1.**
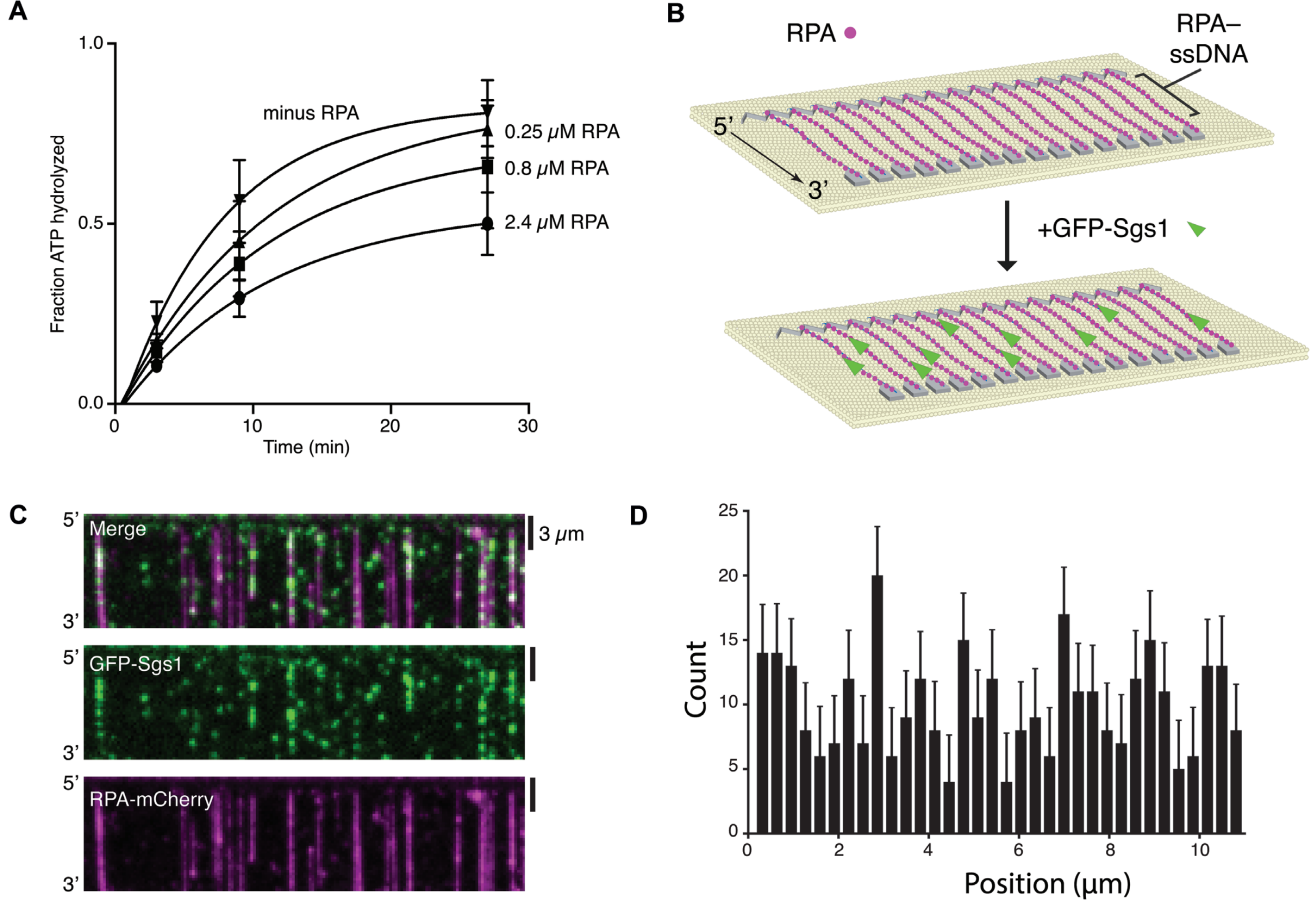
Sgs1 binds to RPA-coated ssDNA. (**A**) ATP hydrolysis assays with 0, 0.25, 0.8, 2.4 μM RPA with unlabeled Sgs1. The data points represent the mean and standard deviation of three independent experiments. (**B**) Schematic of ssDNA curtain assay used to measure the binding and translocation activity of GFP–Sgs1 on ssDNA-RPA molecules. (**C**) Widefield images showing a ssDNA bound by RPA–mCherry (magenta) and GFP–Sgs1 (green). (**D**) Binding distribution of GFP–Sgs1 on ssDNA bound by RPA–mCherry molecules, error bars were generated by bootstrapping the data using a custom python script (*N* = 340).

**Figure 2.**
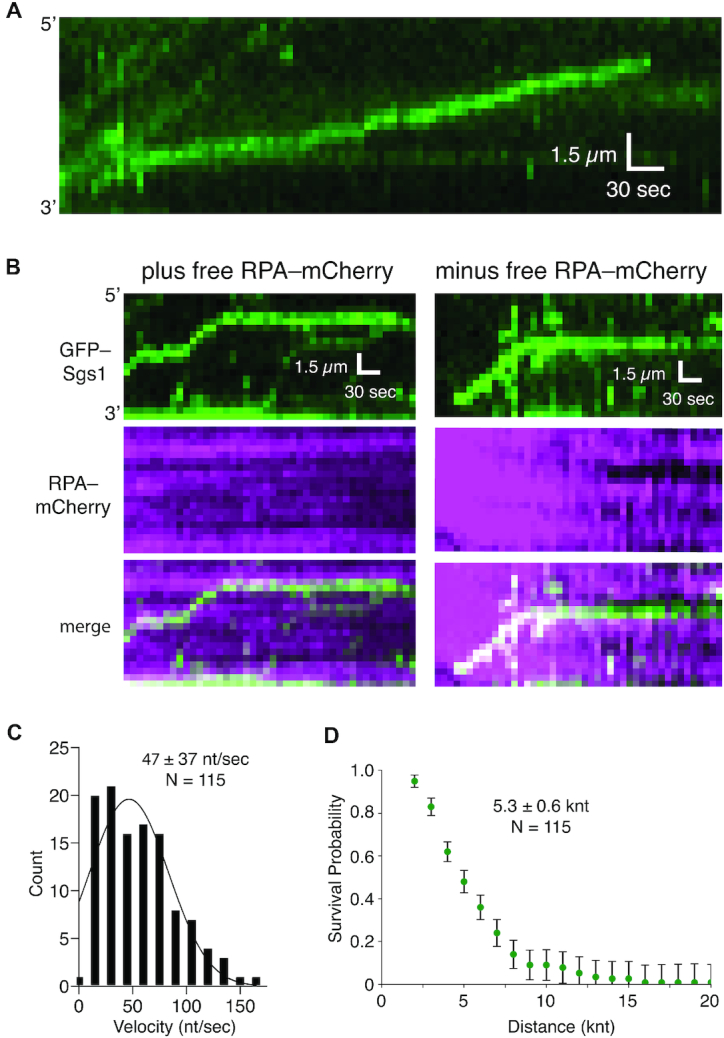
Sgs1 is a robust ssDNA motor protein. (**A**) Representative kymograph of GFP–Sgs1 translocation on unlabeled RPA–ssDNA. (**B**) Representative kymographs illustrating the translocation of GFP–Sgs1 (green) on ssDNA bound by RPA–mCherry molecules in the presence and absence free 0.1 nM RPA–mCherry, as indicated. (**C**) Velocity distribution of individual GFP–Sgs1 complexes translocating on RPA–ssDNA (*N* = 115); the data represents combined results taken from experiments with RPA–mCherry and unlabeled RPA. The data fit a Gaussian distribution and the mean was determined from the fit. (**D**) Survival plot used to determine the processivity of GFP–Sgs1 (*N* = 115); the data represents combined results taken from experiments with RPA–mCherry and unlabeled RPA. Error bars were generated by resampling the data by bootstrapping using a custom python script. All reported processivity values were determined from point in the graph at which the survival probability was equal to 0.5.

Single stranded DNA is a central intermediate in all aspects of DNA replication and repair. However, naked ssDNA is unlikely to exist in physiological settings, instead it quickly becomes bound by the abundant heterotrimeric protein complex RPA (11,18). For instance, during HR, RPA-coated ssDNA is present after DSB end resection and forms a platform for assembling subsequent HR intermediates, and RPA–ssDNA is also core component of the eukaryotic replisome (11,18,31,32). However, it is not known whether Sgs1 can act upon RPA-bound ssDNA. Importantly, N-terminally tagged YFP-Sgs1 forms DNA repair foci *in vivo* and complements a *Δsgs1* strain (33) and strains expressing C-terminally tagged Rfa1-CFP are viable (whereas that deletion of *RFA1* is lethal) and Rfa1-CFP also forms DNA repair foci (34). Therefore, we constructed a GFP–Sgs1 fusion protein for use in single molecule assays to directly visualize Sgs1 activity. We have also previously shown that unlabeled Sgs1 and GFP–Sgs1 exhibit closely comparable dsDNA unwinding activity (with either unlabeled RPA or RPA–mCherry) and GFP–Sgs1 retains the ability to participate in dsDNA end resection (35). Here, we show that GFP–Sgs1 and unlabeled Sgs1 exhibited similar levels of ssDNA-dependent ATP hydrolysis *in vitro* (Supplementary Figure S1A). Interestingly, RPA reduced Sgs1 ATP hydrolysis activity, suggesting that RPA may hinder the motor activity of Sgs1 relative to naked ssDNA (Figure 1A & Supplementary Figure S1B). We next used ssDNA curtain assays to visualize the behavior of Sgs1 at the single molecule level (Figure 1B). These assays revealed that GFP–Sgs1 bound to random sites on ssDNA molecules that were coated with RPA–mCherry (Figure 1C and D). Moreover, we could readily observe 3′→5′ motor activity for GFP–Sgs1 bound to either unlabeled RPA–ssDNA (Figure 2A) or RPA–mCherry-ssDNA (Figure 2B). GFP–Sgs1 exhibited a mean velocity of 47 ± 37 nucleotides per second (nt/s; N = 115) (Figure 2C) and translocated an average distance of 5.2 ± 0.6 kilonucleotides (knt) (*N* = 115) prior to stopping (Figure 2D). We combined the Sgs1 velocity data with labeled and unlabeled RPA in Figure 2D, however, when considered separately there was no statistically significant difference between GFP–Sgs1 translocation for the unlabeled RPA (46 ± 35; *N* = 58) and RPA–mCherry (50 ± 33; *N* = 45) data sets (*P* = 0.36, Student t-test; Supplementary Figure S1C). These experiments demonstrate that GFP–Sgs1 is a motor protein that can translocate on RPA–ssDNA.

Surprisingly, we found no evidence that Sgs1 could remove RPA from the ssDNA (Figure 2B). This result is in direct contrast to Srs2, which readily displaces RPA from ssDNA (22). Interestingly, similar findings have been reported for the archaeal SF2 helicase XPD, suggesting that the ability to co-exist with ssDNA-binding proteins may be a common feature of SF2 family members (36,37). We infer that RPA may maintain constant contact with the ssDNA during the passage of Sgs1, perhaps using a mechanism resembling how nucleosomes and polycomb group proteins remain bound to DNA during transcription and replication, respectively (38).

### Disruption of Rad51–ssDNA filaments by Sgs1

Rad51 catalyzes DNA strand invasion during HR (11,18,31,32). Rad51 is also targeted to replication intermediates to promote HR-dependent rescue of stalled or collapsed forks (11,18,31,32). In yeast, the antirecombinase Srs2 strips Rad51 from ssDNA, which helps prevent aberrant or crossover recombination events (12,20,21,39–42). Importantly, Srs2 and Sgs1 exhibit partial genetic redundancy as, for instance, Sgs1 overexpression suppresses some of the defects of *srs2Δ* cells, and *srs2Δ sgs1Δ* double mutants are synthetic lethal, suggesting they may have overlapping functions (13,19–21). However, it remains unknown whether Sgs1 can act upon Rad51–ssDNA.

**Figure 3.**
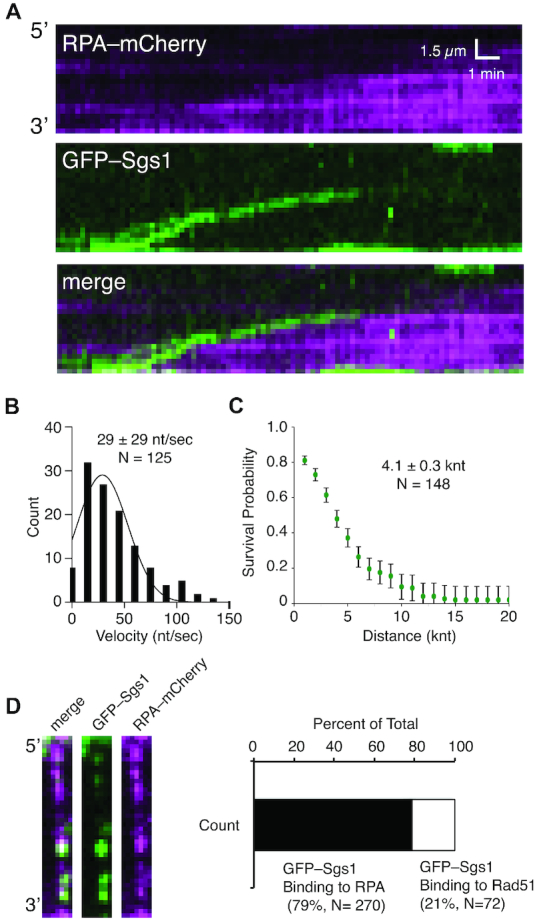
Disruption of Rad51 filaments by Sgs1. (**A**) Representative kymograph showing GFP–Sgs1 (green) translocation on an ssDNA molecule bound by unlabeled Rad51. Rad51 displacement is revealed by rebinding of RPA–mCherry (magenta). (**B**) Velocities distribution for individual Sgs1 translocation events; the data represents combined results taken from experiments with GFP–Sgs1 and unlabeled Sgs1 (*N* = 121). The data fit a Gaussian distribution and the mean was determined from the fit. (**C**) Survival probability plot used to determine the processivity of Sgs1 on Rad51–ssDNA (*N* = 121); error bars were generated by bootstrapping. (**D**) Images of individual Rad51–ssDNA filaments showing embedded RPA–mCherry clusters (magenta) and bound by GFP–Sgs1 (green). (**E**) Graph quantifying GFP–Sgs1 binding locations on Rad51–ssDNA (*N* = 342).

Rad51 had no appreciable impact upon ssDNA-dependent ATP hydrolysis activity of Sgs1 (Supplementary Figure S2A). Rad51 filaments bound to ssDNA in our DNA curtain assays are highly stable and they do not spontaneously disassemble unless ATP is removed from the buffer (24,43,44). Single-molecule experiments performed with wild-type Rad51, wild-type Sgs1 and GFP-RPA revealed that Sgs1 removed Rad51 from ssDNA (Supplementary Figure S2C and D). Moreover, Rad51 displacement occurred in long tracts, suggesting that Rad51 removal was due to processive 3′→5′ translocation activity of Sgs1 (Supplementary Figure S2D). Two-color single-molecule imagining using unlabeled Rad51, GFP–Sgs1 and RPA–mCherry confirmed that Sgs1 translocated along the ssDNA while removing Rad51 (Figure 3A). Sgs1 translocation occurred exclusively in the 3′→5′ direction (Figure 3A) and cumulative data for labeled and unlabeled Sgs1 yielded a mean velocity of 29 ± 29 nt/s (*N* = 125;) and a mean processivity 4.1 ± 0.3 knt (*N* = 148; Figure 3B and C). Interestingly, GFP–Sgs1 was able to translocate on ssDNA bound by human RPA, but it was unable to translocate on ssDNA bound by human RAD51 (Supplementary Figure S3). Together, these data demonstrate that Sgs1 specifically evicts yeast Rad51 from ssDNA.

### Sgs1 recruitment to Rad51 filaments

Two-color experiments using GFP–Sgs1, RPA–mCherry and wild-type Rad51 indicate that the binding of Sgs1 was not random. Instead, GFP–Sgs1 binding events strongly co-localized with clusters of RPA–mCherry embedded between adjacent Rad51 filaments (Figure 3D). Indeed, 79% of all GFP–Sgs1 binding events (N = 270/342) co-localized with RPA–mCherry (Figure 3D). These data suggest that the random binding distributions (when examined at the population level) observed for GFP–Sgs1 (Supplementary Figure S2D) reflected the underlying random distribution of RPA–mCherry clusters embedded between Rad51 filaments (23). A smaller fraction of GFP–Sgs1 binding events (21%, *N* = 72/342) did not coincide with RPA–mCherry (Figure 3D). We cannot rule out the possibility that these binding events may have coincided with small clusters of RPA–mCherry that may have either photobleached or been too small to detect under the illumination conditions used for these experiments. Taken together, these results support a model in which Sgs1 is recruited to RPA clusters present in-between Rad51 filaments.

### Sgs1-mediated disruption of Rad51-I345T nucleoprotein filaments

**Figure 4.**
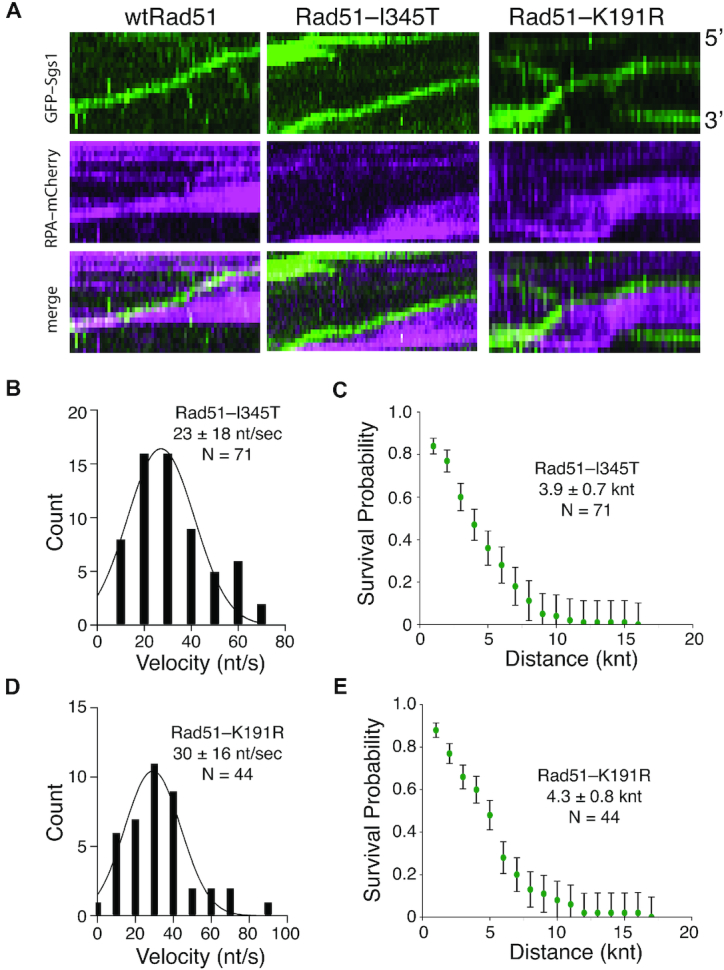
Removal of Rad51 mutants by Sgs1. (**A**) Kymographs illustrating GFP–Sgs1 (green) translocation on ssDNA bound by either wild-type Rad51 (left), Rad51^I345T^ (middle) or Rad51^K191R^-ssDNA (right) in the presence of RPA–mCherry (magenta). (**B**) Velocities distributions for GFP–Sgs1 on Rad51^I345T^-ssDNA (*N* = 70). (**C**) Survival probability plot for GFP–Sgs1 on Rad51^I345T^-ssDNA (*N* = 70). Error bars were generated by resampling the data by bootstrapping using a custom python script. (**D**) Velocities distribution for GFP–Sgs1 on Rad51^K191R^-ssDNA (*N* = 44). (**E**) Survival probability for GFP–Sgs1 on Rad51^I345T^–ssDNA (*N* = 70). Error bars were generated by resampling the data by bootstrapping.

Rad51^I345T^ was isolated as a suppressor mutation that partially bypasses the requirement for the Rad51 paralog complex Rad55–Rad57, suggesting that Rad51^I345T^ may have an increased affinity for ssDNA (45). Consistent with this genetic observation, Rad51^I345T^ assembles into filaments more quickly than wild-type Rad51 and yields more stable filaments (24). Furthermore, nucleofilaments of Rad51^I345T^ are more resistant to disruption by Srs2, which is reflected as a ∼40% reduction in Srs2 translocation velocity (24). We sought to determine whether Rad51^I345T^ might also be more resistant to Sgs1. Interestingly, GFP–Sgs1 readily removed Rad51^I345T^ from ssDNA (Figure 4A), yielding mean velocity and processivity values of 23 ± 18 nt/s and 3.9 ± 0.7 knt (*N* = 70), respectively (Figure 4B and C). These values were statistically indistinguishable from GFP–Sgs1 reactions performed with wild-type Rad51 (*P* = 0.19), indicating that the I345T mutation has no impact upon the ability of Sgs1 to strip Rad51^I345T^ from ssDNA.

### ATP hydrolysis by Rad51 is not necessary for Sgs1-mediated filament disruption

Rad51 requires ATP to bind ssDNA, and ATP hydrolysis and ADP + P_i_ release allows Rad51 to dissociate from ssDNA (18). A mutation in the Walker A box of Rad51 (Rad51^K191R^) greatly attenuates ATP hydrolysis and slows Rad51 dissociation from ssDNA (24,46). Srs2 disrupts Rad51 filaments by stimulating Rad51 ATP hydrolysis activity (39), as a consequence, Rad51^K191R^, which is competent for DNA binding but attenuated for ATPase activity, drastically impairs Srs2 antirecombinase activity, resulting in a ∼75% reduction in translocation velocity (24). Remarkably, GFP–Sgs1 readily removed Rad51^K191R^ from ssDNA (Figure 4A), yielding mean velocity and processivity values of 34 ± 16 nt/s and 4.2± 0.8 knt (*N* = 44), respectively (Figure 4D and E). These values were statistically indistinguishable from Sgs1 assays with wild-type Rad51 (*P*-value = 0.05). Taken together, these findings indicate that Sgs1 can efficiently remove Rad51 from ssDNA even when ATP hydrolysis by Rad51 is not possible.

### Dmc1 inhibitors the motor activity of Sgs1

Dmc1 is a member of the Rad51/RecA recombinase family and is expressed only during meiosis (47–49). Srs2 cannot remove Dmc1 from ssDNA *in vitro* (30), and Srs2 overexpression in meiosis disrupts Rad51 filaments, but leaves Dmc1 foci intact (50). The ability of Dmc1 to inhibit Srs2 may play a role in up-regulating the efficiency of crossover formation during meiosis by preventing the premature dissolution recombination intermediates bound by Dmc1. Like Srs2, Sgs1 is also important negative regulator of crossover formation (12,19). Remarkably, Dmc1 strongly inhibited ssDNA-dependent ATP hydrolysis activity of Sgs1 (Supplementary Figure S4A and B). Consistent with these results, ssDNA curtain assays revealed that Sgs1 could not remove Dmc1 from ssDNA (Supplementary Figure S4C). We conclude from these results that Sgs1 is unable to act upon ssDNA intermediates bound by Dmc1.

### Dmc1 blocks Sgs1 access to ssDNA

**Figure 5.**
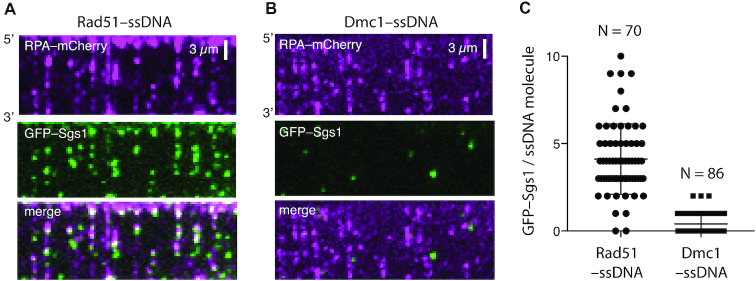
Dmc1 prevents Sgs1 from binding to ssDNA. (**A**) Two-color widefield images of a Rad51–ssDNA (unlabeled) curtain assembled in the presence of RPA–mCherry (magenta). The contrast of these images has been adjusted to highlight the presence of the RPA–mCherry clusters above background. (**B**) Two-color TIRFM widefield images of a Dmc1–ssDNA (unlabeled) curtain assembled in the presence of RPA–mCherry (magenta). The contrast of these images matches the contrast shown in panel A and has been adjusted to highlight the presence of the RPA–mCherry clusters. (**C**) Quantification of the number of GFP–Sgs1 binding events per ssDNA molecule for Rad51–ssDNA (*N* = 70) and Dmc1–ssDNA (*N* = 86). Error bars represent the mean and standard deviation of the data set.

There are two plausible models that could explain why Dmc1 is resistant to Sgs1: Dmc1 could prevent Sgs1 from binding to the ssDNA; or Dmc1 might allow binding but block Sgs1 translocation. These two models are not mutually exclusive, so one can envision a scenario in which Dmc1 inhibits both Sgs1 binding and Sgs1 translocation. To help distinguish between these possibilities, we tested GFP–Sgs1 on Dmc1–ssDNA filaments. We were able to readily visualize GFP–Sgs1 binding to Rad51–ssDNA filaments (Figure 5A), but we detected little or no Sgs1 binding to Dmc1–ssDNA under the same conditions (Figure 5B). As indicated above, the GFP–Sgs1 strongly co-localized with RPA–mCherry clusters embedded in-between Rad51 filaments (Figures 3D & 5A). In striking contrast, we found little or no evidence for GFP–Sgs1 co-localization with RPA–mCherry on Dmc1–ssDNA filaments (Figure 5B). Of the small number of GFP–Sgs1 molecules were bound to the Dmc1–ssDNA filaments, none exhibited evidence of translocation activity on the Dmc1–ssDNA. Quantification of the resulting data revealed a ≥10-fold reduction in the amount of GFP–Sgs1 bound to Dmc1–ssDNA compared to Rad51–ssDNA (Figure 5A–C). We conclude that Dmc1 downregulates Sgs1 activity primarily by preventing Sgs1 from associating with Dmc1-bound ssDNA, but can also block the translocation activity in the rare instances in which Sgs1 binds to the Dmc1–ssDNA.

### Attenuation of Sgs1 translocation velocity by Top3–Rmi1

**Figure 6.**
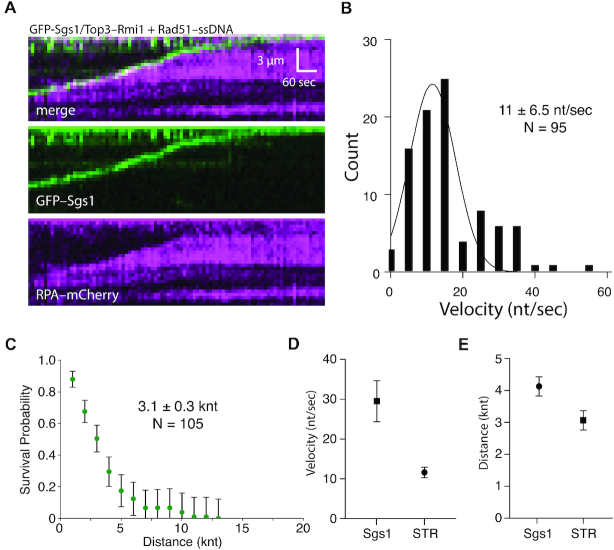
Top3–Rmi1 slows Sgs1 translocation on Rad51–ssDNA. (**A**) Kymograph showing GFP–Sgs1(green)/Top3–Rmi1 translocation on Rad51–ssDNA in the presence of RPA–mCherry (magenta). **(B)** Velocity distribution for GFP–Sgs1/Top3–Rmi1 on Rad51–ssDNA (*N* = 95). (**C**) Survival probability plot for GFP–Sgs1/Top3–Rmi1 on Rad51–ssDNA (N = 105); error bars were generated by resampling the data by bootstrapping using a custom python script. **(D)** Comparison of GFP–Sgs1 translocation velocity with and without Top3–Rmi1 (*P*-value ≤ 0.0001). Error bars represent the 95% confidence interval for the mean of the Gaussian distribution. (**E**) Comparison of the processivity values for GFP–Sgs1 with and without Top3–Rmi1; the difference between the processivity values is not statistically significant (*P*-value = 0.0008). Error bars represent the 95% confidence interval for the half-life of exponential decay function by which the data was fit.

In cells, Sgs1 associates with Top3 (topoisomerase III) and Rmi1 (RecQ-mediated genome instability), forming the STR (Sgs1–Top3–Rmi1) complex (1,11,18). We next asked whether Top3–Rmi1 would alter the ssDNA translocation characteristics of Sgs1. The addition of Top3–Rmi1 caused small reductions in Sgs1 ATP hydrolysis activity with naked ssDNA, RPA–ssDNA or Rad51–ssDNA (Supplementary Figure S5). However, there was no statistically significant change in the velocity or processivity of the STR complex while acting on RPA–ssDNA compared to reactions with Sgs1 alone (*P* value = 0.055; cf. Figure 2 and Supplementary Figure S6). The STR complex could also clear Rad51 from ssDNA (Figure 6A). However, there was a 3-fold reduction in velocity on Rad51–ssDNA (*P* value ≤ 0.0001; Figure 6B and D), as well as a modest reduction in processivity for the STR complex compared to the Sgs1 alone (*P* value = 0.0008; Figure 6C and E). Interestingly, the slow growth, hypersensitivity to DNA damage hyper-sensitivity, and hyper-recombination phenotypes of *top3Δ* cells are all suppressed by deletion of *SGS1* (51). One possible explanation for these findings is that Top3–Rmi1 fine tunes the velocity of Sgs1 to match cellular needs, such that unrestrained Sgs1 activity caused by the absence of Top3 may lead to the aberrant disruption of replication and/or recombination intermediates.

## DISCUSSION

Here, we provide evidence suggesting a new regulatory role for the *S. cerevisiae* RecQ helicase Sgs1 in attenuating the stability of Rad51–ssDNA filaments during homologous recombination and have defined the mechanistic basis for this regulatory activity. We propose that this antirecombinase activity of Sgs1 may reflect its ability to protect stalled or collapsed replication forks from forming toxic recombination-dependent DNA structures. Moreover, our results regarding the inability of Sgs1 to affect the stability of Dmc1–ssDNA filaments have implications for understanding the basis of crossover regulation in meiosis.

### Antirecombinase activity of Sgs1

**Figure 7.**
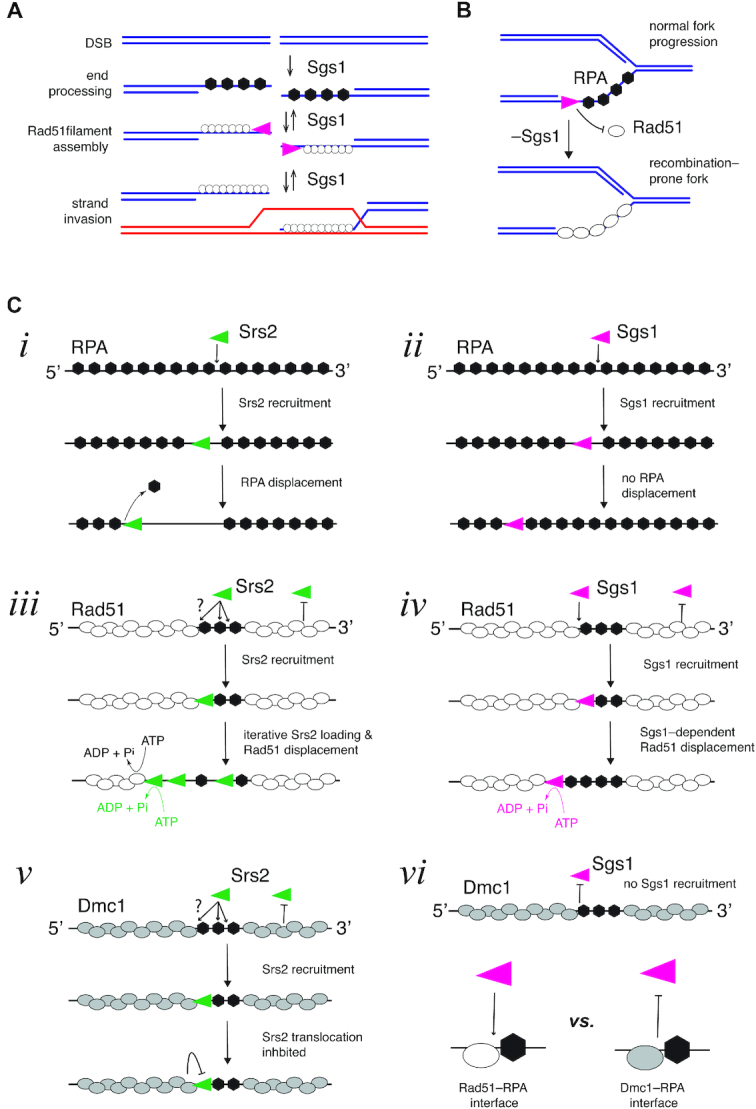
Molecular mechanisms of Sgs1 action on ssDNA intermediates. (**A**) Sgs1 can act at all early stages of HR, and its antirecombinase activity would act similarly to Srs2 by channeling intermediates towards the SDSA pathway of repair. (**B**) Surveillance of RPA–ssDNA may allow Sgs1 to inappropriate accumulation of Rad51 at replication forks, which may otherwise give rise to replication-coupled hyperrecombination. (**C**) Comparison of Srs2 and Sgs1 activities on ssDNA. Srs2 and Sgs1 both translocation on RPA–ssDNA, but (*i*) Srs2 strips RPA from ssDNA, whereas (*ii*) Sgs1 does not. Srs2 and Sgs1 both load at RPA clusters present at the ends of Rad51 filaments, for Srs2 (*iii*) multiple loading events take place and Rad51 removal is coupled to the Rad51 ATP hydrolysis cycle. In the case of (*iv*) Sgs1, loading does not involve iterative binding events, and Rad51 removal is uncoupled from the Rad51 ATP hydrolysis cycle. Neither Srs2 nor Sgs1 can remove Dmc1 from ssDNA, but (*v*) Srs2 inhibition occurs primarily by blocking translocation, whereas (*vi*) Sgs1 is blocked from binding.

Rad51 promotes the DNA transactions that take place during the early phases of HR, and as such represents an important target for regulatory control. Helicase-mediated disruption of recombinase filaments is a common theme in recombination regulation and has been established for both prokaryotic helicases, such as UvrD and PcrA, which act upon RecA filaments (52–54), and also for eukaryotic helicases, such as yeast Srs2, human BLM and human RECQ5, which dismantle Rad51 filaments (2,3,6). Although it is well-known that Sgs1 functions in DSB end processing, D-loop disruption and dissolution of double Holliday junctions (18), genetic evidence for its participation in other aspects of recombination has proven more difficult to establish due to the pleiotropic phenotypes of *sgs1* mutants. Our work now shows that Sgs1 is also capable of evicting Rad51 from ssDNA via its 3′→5′ ssDNA translocase activity (Figure 7A). This mode of action is very similar to that of the antirecombinase Srs2, albeit with several important mechanistic differences (see below).

Our finding that Sgs1 possesses antirecombinase activity can explain some of the molecular defects seen in *sgs1Δ* mutants. Importantly, *sgs1Δ* mutant cells experience severe replication stress that gives rise to aberrant X-shaped replication-dependent recombination intermediates (55–58). This phenotype can be suppressed by either Rad51 deletion or by Srs2 overexpression. The identity of these aberrant replication structures remains uncertain, but they are sensitive to ssDNA-specific nucleases and they coincide with prominent RPA nuclear foci, indicating that they harbor a substantial amount of ssDNA (55,56). Together with these published results, our findings support a model in which the antirecombinase activity of Sgs1 helps protect genomic integrity during S-phase by mitigating recombination-induced DNA replication stress through its ability to prevent inappropriate Rad51 accumulation on replication forks (Figure 7B).

### Sgs1 and Srs2 act through distinct mechanisms

Comparison of Srs2 and Sgs1 may provide insights into the functional differences between these antirecombinases (Figure 7C). For example, Sgs1 and Srs2 both translocate on RPA–ssDNA, although Srs2 displaces RPA whereas Sgs1 does not (Figure 7C); both helicases remove Rad51 from ssDNA, albeit through different mechanisms (*see below;* (Figure 7C); they are both recruited to RPA clusters; and they are both inhibited by Dmc1, but through distinct mechanisms (see below; Figure 7C). However, Srs2 is ∼4-times faster and ∼4-times more processive than Sgs1 while acting on Rad51–ssDNA. Srs2 also undergoes a highly efficient iterative loading process allowing multiple helicase molecules to act collectively on Rad51–ssDNA filaments (24). In contrast, we find little or no evidence for iterative Sgs1 loading (Figure 7C). These considerations suggest that in general Srs2 is likely more adept than Sgs1 at disrupting Rad51 filaments, which is consistent with the primary role of Srs2 being to remove Rad51 from ssDNA, whereas Sgs1 must also perform many additional functions. Interestingly, there are two scenarios where Sgs1 outperforms Srs2 with respect to the removal of Rad51 from ssDNA. Specifically, Srs2 is greatly inhibited by Rad51 K191R, which is deficient for ATP hydrolysis, and is also inhibited by Rad51-1345T, which binds faster and more tightly to ssDNA compared to wild-type Rad51. In contrast, Sgs1 readily removes either of these Rad51 mutants from ssDNA. The findings further buttress our premise that Srs2 and Sgs1 employ distinct mechanisms to execute their antirecombinase functions.

### Sgs1 is recruited to the 3′ end of Rad51 filaments

Sgs1 is loaded at RPA clusters between adjacent Rad51 filaments (Figure 7C). The loading mechanisms with respect to the spatial distribution of RPA appear to be similar for Sgs1 and Srs2 (Figure 7C) (24). We anticipate that this mechanism ensures appropriate regulation of filament disassembly dynamics by confining the actions of antirecombinases ends of the Rad51 filaments. Given that Srs2 and Sgs1 both translocate in the 3′→5′ direction, they would be expected to encounter the ends of the Rad51 filaments that are oriented in the 3′ direction relative to the ssDNA. This filament end-dependent recruitment mechanism also offers the potential for HR accessory factors to regulate the activity of antirecombinases by capping the 3′ ends of the Rad51 filaments. In principle, helicase recruitment could occur via protein–protein interactions with RPA, interactions with the ssDNA, or interactions with the ends of the Rad51 filaments. However, we note that Srs2 is recruited to RPA clusters within Dmc1 filaments (30), whereas Sgs1 is not (Figure 7C). This result implies that Sgs1 recognizes a unique feature of RPA clusters within Rad51 filaments that is absent in Dmc1 filaments - the likely target is the 3′ ends of the Rad51 filaments. Overall, our data are most consistent with a model where Sgs1 is recruited to the 3′ ends of the Rad51 filament, most likely through protein–protein interactions with Rad51 amino acids that would otherwise be buried at the Rad51–Rad51 subunit interfaces. Our model also posits that the requisite interaction is absent in Dmc1 filament ends (Figure 7C).

### Sgs1 mechanism of filament disruption

Rad51 affinity for DNA is linked to its ATP hydrolysis cycle: Rad51-ATP binds tightly to DNA, whereas Rad51-ADP has a much lower affinity for DNA, such that ATP hydrolysis allows for Rad51 dissociation from DNA (18). The Rad51^K191R^ mutation allows ATP binding, but greatly attenuates ATP hydrolysis, and as a consequence also slows protein dissociation from DNA (24,46). Similarly, non-hydrolysable ATP analogs such as AMP-PNP or ATPγS slow or prevent dissociation of Rad51 from DNA (18). Importantly, Srs2 takes advantage of the Rad51 ATP hydrolysis cycle to provoke Rad51 dissociation. The current model postulates that through direct protein–protein contacts, Srs2 stimulates the ATP hydrolysis activity of Rad51 to trigger its dissociation from ssDNA (39). Consistent with this model, the ATP hydrolysis deficient mutant Rad51^K191R^ drastically slows Srs2, corresponding to a ∼75% reduction in Srs2 translocation velocity on ssDNA by Rad51^K191R^ (24,39). Thus, a key feature of the Srs2 antirecombinase mechanism is that it exploits the relationship between Rad51 DNA-binding affinity and its nucleotide-bound state rather than simply displacing Rad51 from ssDNA (24,39). In contrast to Srs2, our work reveals that Sgs1 antirecombinase activity is independent of the Rad51 ATP hydrolysis cycle. As a result, Sgs1 readily removes Rad51^K191R^ from ssDNA with no measurable reduction in translocation velocity. This observation is most consistent with a displacement mechanism where Sgs1 uses the free energy derived from ATP hydrolysis to actively disrupt the contacts that Rad51 makes with ssDNA (Figure 7C).

### Inhibition of Sgs1 antirecombinase activity by Dmc1

Dmc1 and Rad51 emerged from a gene duplication event during the early evolutionary history of eukaryotes (59,60). These two recombinases retain ∼46% amino acid identity, assemble into structurally similar nucleoprotein filaments and both proteins catalyze DNA strand exchange (47,48). Despite their similarities, we find that Sgs1 readily dismantles Rad51 filaments, but is unable to act upon Dmc1–ssDNA filaments. Likewise, Srs2 cannot disrupt Dmc1 filaments (30,50). While crossover recombination events are essential for chromosome segregation during meiosis, they are down-regulated during mitotic growth to minimize chromosomal rearrangements (47–49). The finding that Dmc1 inhibits both Srs2 and Sgs1 suggests that Dmc1 may help channel recombination intermediates away from the SDSA pathway, which would only give rise to non-crossovers, and instead directs these intermediates towards the formation of double Holliday junctions, which can allow for crossover formation. In addition, our finding that antirecombinases responsible for preventing aberrant formation of Rad51 filaments at replication forks are inactive towards Dmc1, suggest that Dmc1 filaments formed on replication intermediates may compromise genome integrity. In this regard, it is interesting to note that mis-expression of Dmc1 in mitotic cells is encountered in human glioblastoma cell lines and accompanied by heightened replication stress (61).

### Human RECQ helicases

Human BLM and RECQ5 can both remove human RAD51 from ssDNA (62,63), which together with our Sgs1 results, suggests that Rad51 filament disruption is a conserved function of some RecQ helicases. However, BLM differs from both Sgs1 and RECQ5 in that while it can displace the ADP-bound form of RAD51 from ssDNA, it is inactive toward RAD51 filaments associated with ATP (62,63). Indeed, we detect no evidence for GFP-tagged BLM interactions with either RPA–ssDNA or RAD51-ssDNA in our DNA curtain assays, even though GFP-BLM exhibits highly processive helicase activity on dsDNA (our unpublished results). One possible inference from these observations is that BLM may not play a significant role in dismantling RAD51 filaments in cells, and that this function has instead been co-opted by RECQ5 and the F-box containing helicase FBH1 (62,64). Alternatively, BLM may be subject to additional layers of regulatory control (e.g. post-translational modifications or interactions with partner proteins) in order to function as an antirecombinase. This later possibility is consistent with the observation that BLM suppresses RAD51 filament assembly *in vivo* (65).

## Supplementary Material

Supplementary Data
