# Supplementary material for: The RecQ helicase Sgs1 drives ATP-dependent disruption of Rad51 filaments
Source: Nucleic Acids Res. 2019 Mar 27;47(9):4694–706. doi: 10.1093/nar/gkz186 (PMC6511845; doi:10.1093/nar/gkz186)
Supplement: Supplementary Data [file gkz186_supplemental_files.pdf]

## SUPPLEMENTAL FIGURE LEGENDS

**Fig. S1. GFP–tagged Sgs1 is an active ATPase. (A)** Comparison ATP hydrolysis efficiency for wild–type Sgs1 and GFP–Sgs1. Error bars represent the standard deviation of three independent experiments. **(B)** GFP-Sgs1 ATP hydrolysis assays with 0, 0.25 μM, 0.8 μM, 2.4 μM RPA-mCherry. The data points represent the mean and standard deviation of three independent experiments. **(C)** Velocity measurements for individual GFP-Sgs1 traces on RPA-mCherry-ssDNA, WT-RPA-ssDNA, and the combination of both data sets.

**Fig. S2. Wild–type Sgs1 can disrupt Rad51 filaments. (A)** Schematic of experiment to test the effect of Rad51 on the ATP hydorlysis efficiency of Sgs1 and corresponding ATP hydrolysis graph. The ATP hydrolysis activity of wild–type Sgs1 with naked DNA is shown as a dotted green line. The remaining traces show the effect of increasing Rad51 concentrations on the ATP hydrolysis efficiency of Sgs1. Error bars represent the standard deviation of three indepdent experiments. **(B)** Schematic of ssDNA curtains experiment to test the activity of wild–type Sgs1 on Rad51–ssDNA. Here, the dissociation of of Rad51 is revealed by the binding of RPA–GFP. **(C)** Kymograph illustrating the trasnlocation of wild–type Sgs1 in the 3'→5' direction on Rad51–ssDNA as revealed by RPA–GFP. **(D)** Binding site distribution histogram GFP–Sgs1 on Rad51–ssDNA. Error bars are generated by boostrapping with a custom python script.

**Fig. S3. Yeast Sgs1 is unable to remove human RAD51 from ssDNA. (A)** Kymograph depciting the translocation of GFP-Sgs1 on hRPA-mCherry-ssDNA curtains. **(B)** Kymograph depicting the binding of GFP-Sgs1 to hRAD51-ssDNA filaments. Note that GFP-Sgs1 bound to hRAD51-ssDNA filaments, but it did not translocate or otherwise remove Rad51 from the ssDNA.

**Fig. S4. Dmc1 inhbits Sgs1 activty. (A)** Comparison of Sgs1 ATP hydrolysis activty on naked ssDNA and in the presence increasing concentrations of Dmc1. **(B)** Comparison of Sgs1 ATP hydrolysis efficiency in the presence of Rad51 only, Dmc1 only, or 3:1 and 1:1 mixtures of Rad51:Dmc1, as indicated. Error bars represent the standard deviation of three independent experiments. **(C)** Kymograph showing that Sgs1 is unable to remove Dmc1 from ssDNA (see Fig. S2 for comparison to a reaction with Rad51). As a control, 7M urea was quickly injected at the indicated time point to strip Dmc1 from the ssDNA, which was then quickly coated with RPA–GFP.

**Figure S5. Top3–Rmi1 does not affect Sgs1 ATP hydrolysis activty. (A)** Comparison of ATP hydrolysis activity of Sgs1 only (10 nM) and Sgs1 (10nM) plus Top3-Rmi1 (10 nM) on naked ssDNA. **(B)** Comparison of ATP hyrolysis activity of Sgs1 (10 nM) and Sgs1 (10 nM) plus Top3-Rmi1 (10 nM) on RPA–ssDNA. **(C)** Comparison of ATP hydorlysis activity of Sgs1 (10 nM) and Sgs1 (10 nM) plus Top3-Rmi1 (10 nM) on Rad51–ssDNA. For all panels, error bars represent the standard deviation of three independent experiments.

**Figure S6. Translocation of the STR complex on RPA–ssDNA. (A)** Kymograph for illustrating the translocation of GFP-Sgs1/Top3–Rmi1 (the STR complex; shown in green) on ssDNA bound by RPA–mCherry (magenta). **(B)** Velocity for the  the STR complex (N=60) on RPA–ssDNA. The mean velocity and standard deviation were determined from a Gaussian fit to the data. **(C)** Survival plot for the STR complex on RPA–ssDNA (N=60); error bars were generated by bootstrapping.

**Figure S1**

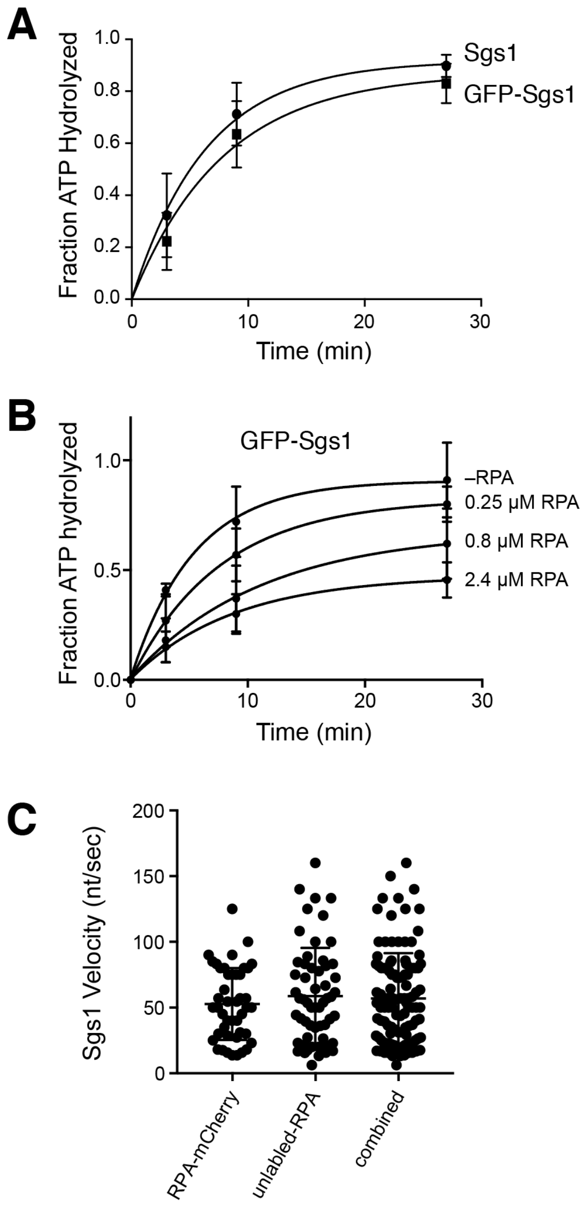
A
Fraction ATP Hydrolyzed
1.0
0.8
0.6
0.4
0.2
0.0
0
10
20
30
Time (min)
Sgs1
GFP-Sgs1
B
Fraction ATP hydrolyzed
GFP-Sgs1
1.0
0.5
0.0
0
10
20
30
Time (min)
–RPA
0.25 µM RPA
0.8 µM RPA
2.4 µM RPA
C
Sgs1 Velocity (nt/sec)
200
150
100
50
0
RPA-mCherry
unlabled-RPA
combined

**Figure S2**

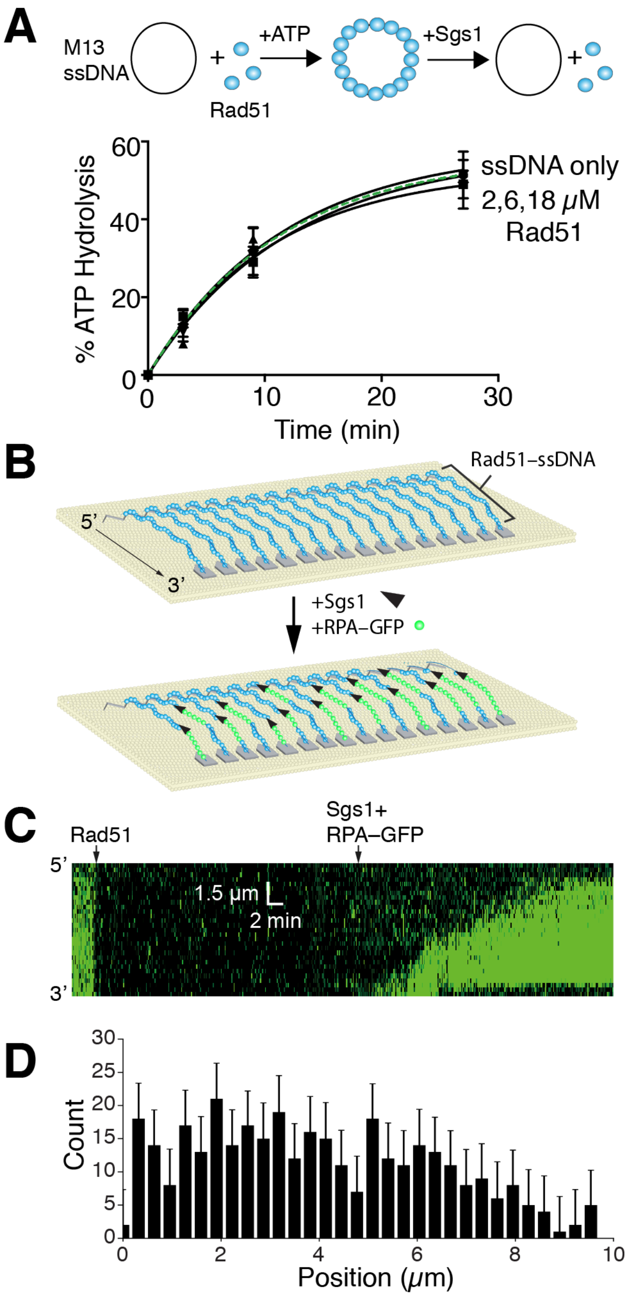
A
M13
ssDNA
Rad51
+ATP
+Sgs1
% ATP Hydrolysis
Time (min)
ssDNA only
2,6,18 μM
Rad51
B
Rad51–ssDNA
5'
3'
+Sgs1
+RPA–GFP
C
Rad51
Sgs1+
RPA–GFP
5'
3'
1.5 μm
2 min
D
Count
Position (μm)

**Figure S3**

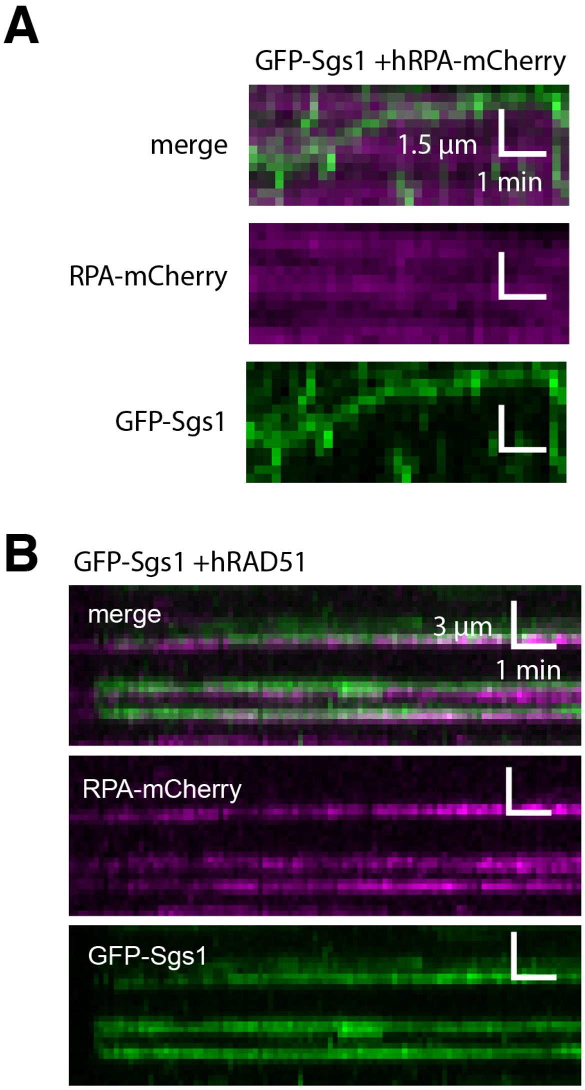
A
GFP-Sgs1 +hRPA-mCherry
merge
1.5 µm
1 min
RPA-mCherry
GFP-Sgs1
B
GFP-Sgs1 +hRAD51
merge
3 µm
1 min
RPA-mCherry
GFP-Sgs1

**Figure S4**

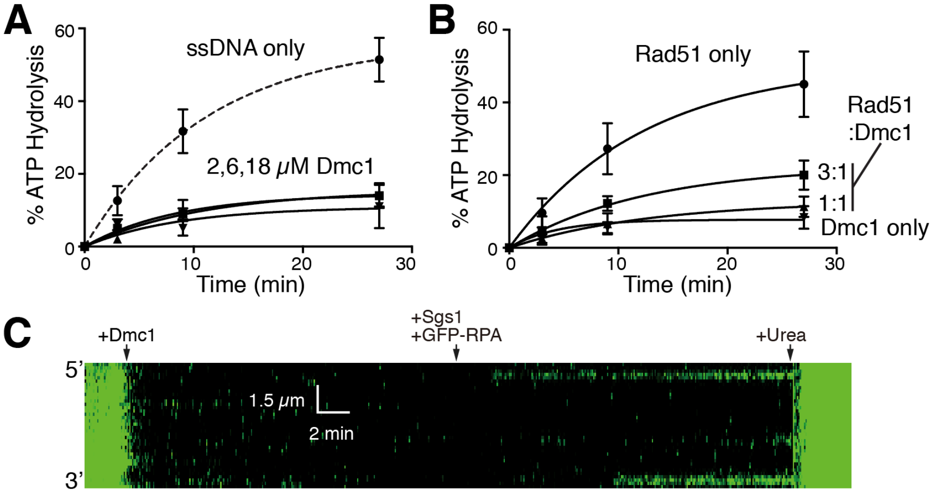
A
B
C
% ATP Hydrolysis
Time (min)
ssDNA only
2,6,18 μM Dmc1
Rad51 only
Rad51
:Dmc1
3:1
1:1
Dmc1 only
+Dmc1
+Sgs1
+GFP-RPA
+Urea
5'
3'
1.5 μm
2 min

**Figure S5**

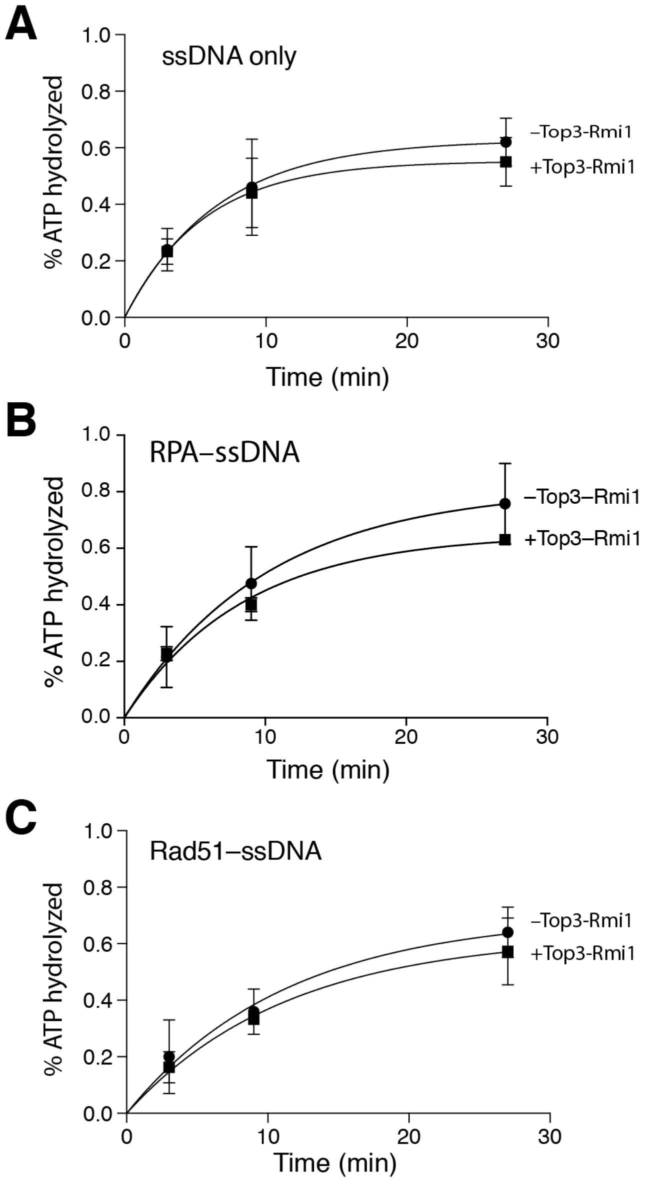
A
ssDNA only
% ATP hydrolyzed
1.0
0.8
0.6
0.4
0.2
0.0
0
10
20
30
Time (min)
−Top3-Rmi1
+Top3-Rmi1
B
RPA−ssDNA
% ATP hydrolyzed
−Top3−Rmi1
+Top3−Rmi1
Time (min)
C
Rad51−ssDNA
% ATP hydrolyzed
−Top3-Rmi1
+Top3-Rmi1
Time (min)

**Figure S6**

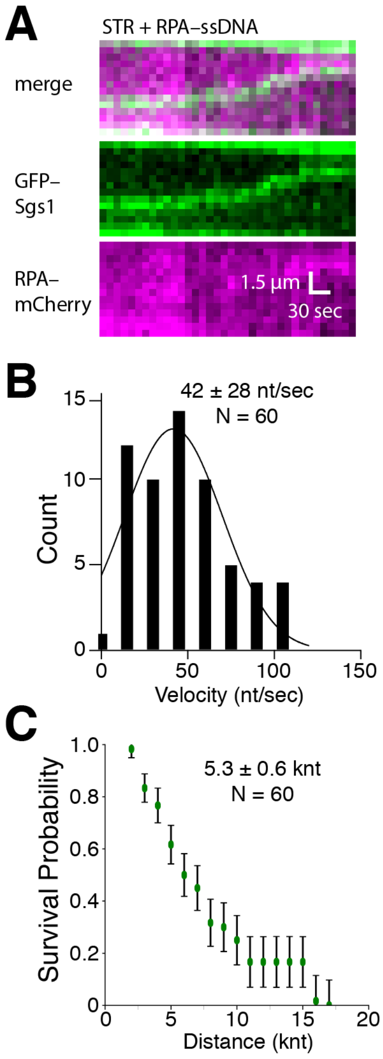
A
STR + RPA–ssDNA
merge
GFP–
Sgs1
RPA–
mCherry
1.5 µm
30 sec
B
42 ± 28 nt/sec
N = 60
Count
Velocity (nt/sec)
C
5.3 ± 0.6 knt
N = 60
Survival Probability
Distance (knt)
